# Linking secondary school physical education with community sport and recreation for girls: a process evaluation

**DOI:** 10.1186/1471-2458-14-1039

**Published:** 2014-10-06

**Authors:** Meghan M Casey, Amanda Telford, Amanda Mooney, Jack T Harvey, Rochelle M Eime, Warren R Payne

**Affiliations:** School of Health Sciences and Psychology, Federation University Australia, Ballarat, Australia; School of Medical Sciences, Discipline of Exercise Sciences, RMIT University, Melbourne, Australia; School of Education, Faculty of Arts & Education, Deakin University, Melbourne, Australia; Institute of Sport, Exercise and Active Living (ISEAL), Victoria University, Melbourne, Australia

**Keywords:** Adolescent girls, School, Community, Physical activity, Sport, Program evaluation, Rural/regional

## Abstract

**Background:**

The purpose of this study was to undertake a process evaluation to examine the reach, adoption and implementation of a school-community linked physical activity (PA) program for girls aged 12 – 15 years (School Years 7 – 9) using the RE-AIM framework.

**Methods:**

Various approaches were used to assess 'reach’, 'adoption’ and implementation: (a) a school environment survey of intervention schools (n = 6); (b) teacher feedback regarding the professional development component (91.1% response rate) and lesson implementation (60.8% response rate); and (c) post-intervention focus group interviews with physical education (PE) teachers (n = 29), students (n = 125), coaches (n = 13) and instructors (n = 8) regarding program experiences.

**Results:**

*Reach and Adoption:* Seven schools (n = 1491 Year 7–9 female student enrolment; 70% adoption rate), five tennis clubs, eight football clubs and five leisure centres participated in the program during 2011. *Implementation:* Program design and professional development opportunities (training, resource manual and opportunities to work with coaches and instructors during PE classes) supported implementation and student engagement in PA. However, there was a lack of individual and organisational readiness to adopt program principles. For some deliverers there were deeply embedded ideologies that were not aligned with the Game Sense teaching approach upon which the program was based. Further, cognitive components of the program such as self-management were not widely adopted as other components of the program tended to be prioritised.

**Conclusion:**

The program design and resources supported the success of the program, however, some aspects were not implemented as intended, which may have affected the likelihood of achieving further positive outcomes. Barriers to program implementation were identified and should be considered when designing school-community linked interventions. In particular, future programs should seek to assess and adjust for organizational readiness within the study design. For example, shared commitment and abilities of program deliverers to implement the program needs to be determined to support program implementation.

**Trial registration:**

ACTRN12614000446662. April 30th 2014.

## Background

The decline in physical activity (PA) levels and the possible associated health problems of young people continue to be of growing concern internationally [1]. The decline in PA levels is greatest during the secondary school years (aged 12–18 years), and previously published research consistently reports lower PA among adolescent girls than among adolescent boys [1, 2]. Globally, an estimated 80% of adolescents (aged 13 – 15 years) are not meeting the PA recommendations of at least 60 minutes of moderate to vigorous PA daily [1]. As a consequence increased attention has been directed towards the development and evaluation of PA interventions targeting young people, and in particular adolescent girls [3–5]. Key settings for PA promotion among adolescents include schools, home, community and primary care; however, by far the most commonly targeted setting for this age group is schools [3, 6].

Schools are considered a key health setting to promote PA since young people attend and spend a considerable amount of time in school [7]. The convenience of targeting existing curricular opportunities like physical education (PE), in addition to extra-curricular opportunities such as sport programs makes schools an obvious setting in which to deliver programs designed to promote PA participation. Numerous PA promotion interventions have been conducted within school settings with varying success [6]. A systematic review of interventions to promote PA within adolescent populations reported that the strongest evidence of success was associated with multicomponent school-based interventions that are combined with community opportunities for PA that address multiple levels of influence on behaviour (i.e. as outlined in socio-ecological frameworks) and include enjoyable PE experiences as a main component [3]. Effective interventions are also reported to consist of both a PA and cognitive component [6]. Peer support strategies are also considered promising; whereas family support has been reported by some as ineffective [3]. Strategies considered most appropriate for public health agencies and partners to promote PA to adolescents include community-wide campaigns, increased access to places in which to be active, educational information or outreach programs, and enhanced PE programs integrated within a socioecological perspective [8].

Most PA promotion interventions have been conducted in the United States and United Kingdom and have tended to focus on children (aged 6–12) rather than adolescents (aged 13 – 19) [6]. They have also have tended to include both sexes [6], despite adolescent girls being identified as a priority group for PA promotion and the need to design interventions to specifically address the needs of girls [3]. Furthermore, few PA interventions for girls have been conducted in low-socioeconomic areas [3], despite adolescents from low income communities having lower levels of PA [9]. As a result, a school-community linked PA-promotion intervention program targeting adolescent girls living in low-SES Australian rural and regional communities was developed, implemented and evaluated. The aim of the program was to improve Health-Related Quality of Life (HRQoL), levels of PA, and a range of potential mediators of PA (e.g. self-efficacy, perceived sport competence). The outcomes of a pre- and post- evaluation of effectiveness of this program found that: 1) The intervention had a protective effect on the intervention group’s health-related quality of life (HRQoL), whereby the intervention group maintained their HRQoL, whilst a decrease was observed in the control group. 2) There were no statistically significant differences between groups for any of the PA measures including - mins of leisure-time moderate-vigorous physical activity (LTMVPA), MET-mins of LTMVPA, or in the proportion meeting PA guidelines. 3) Among 'completers’ — those who had participated in the both the in-school component and outside of school community component — the intervention had positive effects on intra-personal capacities (i.e. self-efficacy, self-management, perceived behavioural control, outcome expectancy-value) and inter-personal factors (i.e. support from family and friends) [10]. Understanding the reach, adoption and implementation of this program is important to help understand why the program was successful in achieving some of the intended outcomes and why other elements were not achieved.

PA intervention studies commonly provide an evaluation of the effectiveness of the intervention, however, more recently there has been a greater emphasis placed upon the importance of evaluating the context in which interventions are implemented [11]. Indeed, few studies have provided a process evaluation of PA interventions targeted at girls [12, 13]. Process evaluation is useful to help understand why a program was successful in achieving its intended outcomes [14]. Process evaluation of multicomponent interventions can be complex and mixed method research designs are required to understand the variety of contexts and settings [13, 15]. The RE-AIM model (reach, effectiveness/efficacy, adoption, implementation and maintenance) provides a systematic framework by which researchers can evaluate the external validity of multicomponent interventions within real-world settings such as schools [16], and has been used to evaluate school-based PA interventions, mostly in primary schools [7, 17, 18] and recently in a secondary school [12].

The aim of this research was to conduct a process evaluation to understand the reach, adoption and implementation of a school-community linked PA intervention for adolescent girls (aged 12 – 15 years) in low-socioeconomic regional and rural areas of Victoria, Australia. Effectiveness of the intervention has been published previously [10] and maintenance was not evaluated due to the short duration of follow-up of this study. Reflection on the methods adopted in the process evaluation of this multicomponent intervention are also discussed to inform future process evaluation plans.

## Method

### Research design

The research was a cluster-randomised trial with participants grouped by schools (intervention and control) and data collected at baseline and endpoint (one year later). As previously outlined [10] the potential pool of intervention and control schools was established using measures of socioeconomic level (Socio-Economic Indexes for Areas Index of Relative Socio-economic Advantage and Disadvantage: SEIFA IRSAD) [19] and isolation (Accessibility and Remoteness Index of Australia: ARIA+) [20], the Victorian Government classification of regions [21], and population size based on the 2006 Australian Bureau of Statistics (ABS) Census. All communities with SEIFA IRSAD below the Victorian median (i.e. 1009), classified by ARIA + as being inner or outer regional, and having a local recreation facility, tennis club and football (soccer) club, were eligible for inclusion. Communities were then matched on population size with one randomly selected community receiving the intervention and the other acting as control. All Victorian rural and regional secondary schools within these matched communities were eligible for inclusion. Schools were then randomly selected from the control (n = 5) and intervention (n = 6) communities and invited to participate, until a total of 16 schools, eight from each of the control and intervention communities, agreed to participate. Tables 1 and 2 provide a summary of the intervention and control school communities. A pseudonym for each community and school was used to protect the identity of participants – e.g. Intervention Community 1 (IC1) and Intervention School A.

**Table 1:** **Summary of intervention community (IC) profile**

Regional town ^a^	Population ^b^	SEIFA	ARIA ^c^	RRMA	Distance from capital city (approx)	School ^a^
IC1	78,222	893	0.459 highly accessible	Large regional centre	115 km	Intervention A*
		993				Intervention B
		993				Intervention C
IC2	13,245	958	2.830 accessible	Other regional town	331 km	Intervention D
IC3	10,438	990	1.310 highly accessible	Small regional centre	105 km	Intervention E
IC4	8,614	918	1.331 highly accessible	Other regional town	213 km	Intervention F
IC5	7,481	885	1.656 highly accessible	Other regional town	170 km	Intervention G
IC6	4,233	972	2.627 accessible	Other regional town	160 km	Intervention H

^a^Denotes pseudonym used to protect anonymity of the community, schools and participants ^b^Data drawn from 2006 ABS Census for state suburb ^c^ARIA values for 1999 Statistical Local Areas (Department of Health and Aged Care, 2001) *withdrew from study.

**Table 2:** **Summary of control community (CC) profile**

Regional town ^a^	Population ^b^	SEIFA	ARIA ^c^	RRMA	Distance from capital city (approx)	School ^a^
CC1	68,716	931	0.598 highly accessible	Large regional	150 km	Control A
		983	0.350 highly accessible	centre		Control B
		983	0.350 highly accessible			Control C
CC2	12,856	983	1.767 highly accessible	Small regional centre	216 km	Control D
CC3	10,953	958	1.347 highly accessible	Small regional	221 km	Control E
		958		centre		Control F
CC4	6,834	942	0.712 highly accessible	Small regional centre	123 km	Control G
CC5	6,152	909	1.459 highly accessible	Other regional town	136 km	Control H

^a^Denotes pseudonym used to protect anonymity of the community, schools and participants ^b^Data drawn from 2006 ABS Census for state suburb ^c^ARIA values for 1999 Statistical Local Areas (Department of Health and Aged Care, [20]).

### Recruitment and participants

Ethics approval was obtained from the University of Ballarat Human Research Ethics Committee, the Department of Education and Early Childhood Development (DEECD), and the Diocese of the Catholic Education Office. Principals of selected intervention and control schools were initially contacted via email, with follow up telephone calls to invite their school to participate and to seek permission to contact the Health and PE (HPE) Department Head. The HPE Department Head was then contacted, informed about the study, and invited to agree to register their school’s participation in the study. The research team held meetings with HPE staff at all intervention schools to explain the purpose of the research, the methods, demands, potential risks, inconveniences and discomforts for staff and students, and possible outcomes of the research. All female students in Years 7 – 9 in control and intervention schools received an information pack that contained a plain language statement (PLS) and consent forms (parental and student) to participate in the study. In collaboration with industry partners (the state peak organizations for tennis, football and YMCA), local tennis clubs, football clubs and YMCA centres were also contacted and invited to participate in the study. Further, face-to-face meetings were held with officials from the identified local sport and recreation organisations to discuss the scope of the intervention and their role in program implementation.

### Intervention program

Details of the program design and implementation instructions have been published previously [22]. In brief, the program consisted of a school PE component which incorporated student-centred teaching approaches and behavioural skill development. It involved students participating in two 6-session units in PE classes – a sport unit (tennis or football) and a recreational unit. Up to three of the PE classes in each unit were delivered in a collaborative manner by PE teachers with the relevant community fitness instructors, and tennis and football coaches, and were linked to a community component that was designed to address previously reported barriers to PA participation. As previously outlined [10] “barriers such as skill level, competence, financial costs and teaching/coaching approaches were identified through ethnographic fieldwork and informed the design of the program [22–24]” (p. 3). The socio-ecological model [25] was the overarching theoretical framework that guided the development of the intervention to help address the wide range of factors which impact upon an individual’s behaviour and is commonly applied to PA interventions [26, 27]. The socio-ecological model was underpinned by self-management strategies based upon Social-Cognitive Theory (SCT) to encourage adolescent girls to be independently active [28] and a capacity-building framework [29] to build the capacity of the teachers and coaches to deliver the program within the schools and community, respectively. Specific capacity building strategies included professional development to introduce the key principles of the planned curriculum and teaching approach. The curriculum and teaching approach drew on the principles of Game Sense [30] and productive pedagogies [31] in curriculum development, which is further described in Casey et al. [22]. Game Sense was adopted as the pedagogical approach for each of the sports units (tennis and football) to focus on the tactical dimensions of the game, rather than skill performance [32], and is the approach commonly adopted in community sports club coaching environments. Productive pedagogies include the dimensions of intellectual quality, connectedness, supportive classroom environment, and working with, and valuing, difference. These were incorporated in classroom practices through teaching and learning cues outlined in the lesson plans [31].

### Evaluation

The RE-AIM framework [33] was used to evaluate the public health impact of the intervention and to examine the barriers and facilitators to the implementation of the program at the school level. Table 3, which was adapted from Janssen et al. [7] summarises the relevant three elements of RE-AIM explored in the process evaluation of the program implemented in the intervention schools, along with a brief description of the outcome measures and methods of assessment associated with the three elements.

**Table 3:** **Reach, adoption and implementation evaluation measures**

Element	Level	Definition [7, 33]	Outcome measure	Method of assessment
Reach	Individual & Organizational	The number and characteristics of participants that receive, or are affected by the intervention.	● Percentage of adolescent girls who received the program in their PE class	● Number of PE classes that received the program (with est. student numbers)
			● Characteristics of the participating population compared to comparable communities	● Compare intervention school to population norms
Adoption	Individual & Organizational	The number of students participating in community program outside of school.	● Attendance at a community program at a tennis club, football club or YMCA/leisure centre	● Post-intervention student survey
		The number and representativeness of schools and intervention staff that adopt the program.	● Organizations that were willing to participate in the program	● Field notes of the number of organizations that participated divided by those who declined (or dropped out) and non-participation reasons
			● Characteristics of participating schools	● Intervention school environments (e.g. enrolments, minutes of PE/week)
Implementation	Organizational	The quality and consistency of delivering the program by schools, clubs and YMCAs and participant satisfaction of the program delivered.	● Qualitative data that examined how the program was implemented and received by students, teachers, coaches and instructors	● Post-intervention Interviews with teachers, coaches, instructors and students and field notes
				● Teacher lesson feedback forms

Reach was defined as the number and characteristics of participants that received, or were affected by the intervention. The number of PE classes that received the program with estimated student numbers is reported. In terms of characteristics of the participating population, data from those students that completed both pre- and post-measures were reported in the effectiveness evaluation of the intervention [10].

Adoption was defined as the participation rate and representativeness of schools (i.e. characteristics of participating schools) that adopted the program. The NSW Schools PA and Nutrition Survey (SPANS) [34] was used to measure the physical environment (e.g. facilities and equipment), school policies (e.g. time allocated for PE and sport) and school practices to promote participation in PA (e.g. making facilities available before/after school or at lunchtime). The reasons why schools declined an invitation to participate as an intervention school were also recorded to understand barriers to program adoption.

Implementation was concerned with the extent that the program was implemented in each of the intervention schools and the level of satisfaction of program deliverers and students with the program. Implementation was measured by:

1. Teacher-reported feedback about the professional development sessions to understand whether the workshop content was relevant and enhanced awareness (or validated their understanding) of the key factors influencing girls participation in PE and community-based sport and PA. Furthermore the feedback was used to understand whether the workshop was useful as a prompt for staff to start planning how the program could be implemented in their school.
2. Teacher-reported session feedback to collect information on each of the sessions. This included information on the year level, class structure, whether a coach/instructor attended the session and whether students were asked to complete the self-management journal entry. Information was also collected using five-point Likert scales on: student response to the session and response to the coach/instructor methods/approach/style (very negatively – very positively); the proportion of the lesson implemented in line with the lesson plan (almost all <80% - hardly any <20%); and the appropriateness of lesson activities, quality of lesson materials, and overall assessment of how the lesson went (poor – excellent).
3. Post-intervention focus group interviews were held with PE teachers (n = 29; approx. 60 minutes/group), students (n = 125; approx. 20–30 minutes/group), coaches (n = 13; approx. 30 minutes/coach) and instructors (n = 8; approx. 60 minutes/instructor) to gain an understanding of the perceptions and experiences of participants during the intervention. For instance, teachers, coaches and instructors were asked to: comment on how students responded and engaged with the program; describe the implementation of the program by commenting on how a typical class of football, tennis or recreational activities were delivered; identify barriers or facilitators to implementing the program; and discuss how well resourced they were when delivering the program in terms of both the intervention resources (e.g. resource manual) and school support and resources. Students were asked to reflect on what they liked and disliked about the program, how the program contributed to their learning or likelihood of adopting PA, and what factors influenced their decision about attending (or not attending) the community-based programs.
4. Personal communication via email correspondence and phone conversations between the research team and program deliverers were recorded as field notes and provided additional information on program implementation.

### Analysis

Qualitative data from the interviews and focus group discussions were transcribed verbatim and were analysed using a constant comparative method, whereby the researcher continually referred to previously coded comments for comparison [35]. Field notes including email correspondence were also recorded, filed by the project manager, and included in the constant comparative method of analysis. Qualitative data were managed and coded using NVivo Version 9 software program [36] to maintain a chain of evidence as recommended [37]. To enhance trustworthiness of the findings several strategies were applied including: 1) data triangulation to build a coherent justification for themes, which involved determining whether themes were present under different research methods (e.g. quantitative and qualitative) and/or reported by different people; 2) rich, thick descriptions to help convey and interpret the findings; and 3) peer debrief to review the findings and ask questions about the research [38].

Quantitative data pertaining to program implementation (e.g. feedback forms and school environment survey) were coded and analysed using standard descriptive statistics such as means, standard deviations, proportions and percentage. All quantitative analyses were conducted using SPSS (Version 18.0).

## Results

### Reach

**Figure 1:**
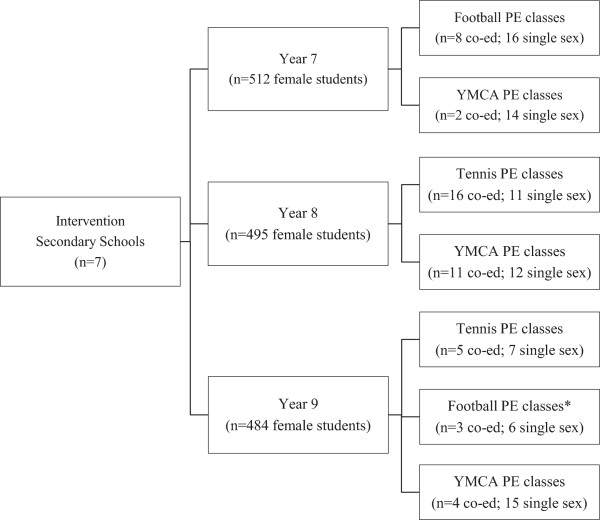
**The number of schools, students and PE classes that received the program.** *One school delivered the program through the Year 9 single sex sport class.

As shown in Figure 1, seven schools completed the program during 2011, where 129 Year 7 – 9 PE classes and one sport class (n = 1491 Year 7 – 9 female student enrolment) participated in the program. The seven schools included classes that were co-educational (n = 49; 37.7%) and single-sex (n = 81; 62.3%). Baseline surveys were returned by 502 students (33.7% recruitment rate) and endpoint surveys were returned by 362 students (61.3% retention or 20.7% overall response rate). At baseline, the mean age of students was 13.4 (±0.9) years and the mean proportion of students meeting PA guidelines in the past seven days was 11.2% [10]. The proportion of students in this study who meet the PA guidelines of at least 60 minutes of MVPA daily is comparable to that reported in a sample of Australian students, whereby 12.7% of students aged 12 – 13 years and 12.1% aged 14 – 15 met the PA guidelines [39].

### Adoption

A total of eight schools responded positively to the invitation to participate in the intervention; whilst two schools declined the invitation. The reasons cited by these two schools for non-participation were having already incorporated a program aimed at improving PA participation for girls in one school, and a concern regarding the impact of the intervention on teacher workload at the other. During program implementation, one of the eight intervention schools (n = 264 Year 7 – 9 student enrolment) withdrew from the program due to unsatisfactory experiences with a community sport and recreation provider, and all data pertaining to this school were excluded from analyses.

In terms of the intervention school characteristics, the schools had a mean student enrolment of 913.3 ± 256.5 with 80.8 ± 23.2 equivalent full-time (EFT) teaching staff, of which 6.8 ± 3.1 EFT staff were PE teachers. The perception of the school sport/PE facilities (2.0 ± 1.3) and equipment (2.2 ± 0.8) was generally 'fair, in need of some improvement’. Only three schools (range: 100 – 200 minutes per week of PE) met the Victorian Government School Sport and PE mandate of 100 minutes per week of PE and sport [40] for all three year levels (Years 7 – 9). Very few schools offered physical activities at lunchtime (33.3%) or after school (33.3%) and none offered physical activities before school.

A total of five tennis clubs, eight football clubs and five leisure centres adopted the program and offered outside-of-school programs that were linked to the school program. One school was not provided with a tennis program outside of school due to the lack of available coaches and/or club personnel. Another school did not receive the leisure centre program due to the lack of capacity of the facility to staff the program.

Generally, all female students in Years 7 – 9 participated in the school component of the program as part of their PE curriculum, with the exception of two schools – one implemented the Year 9 component via a school sport program, and the other only implemented the program in girls-only PE classes. Participation outside-of-school in the community programs was voluntary and required the girls to make their own way to the community venue. A quarter of the intervention group (n = 91; 25.1%) reported attending a community program at a tennis club (n = 28; 7.7%), a football club (n = 28; 7.7%) and/or at a leisure centre (n = 63; 17.4%). The participation rate of the intervention group exceeded both National and State participation rates of females aged 5 – 14 years for football (6.5% and 3.5%) and the National participation rate in tennis (6.3%), although marginally falling short on the State participation rate in tennis (8.2%) [41]. The key reasons for non-attendance at a community program given by those who completed the follow-up survey were that they disliked the activity (n = 35; 9.6%), they had no-one to go with them to the program (n = 31; 8.6%), it was too hard to get home from (n = 29; 8.0%) and/or hard to get to (n = 26; 7.2%) and they had other sport activity commitments (n = 26; 7.2%). Cost as a reason was reported least (n = 19; 5.2%). Discussions with students reinforced many of these barriers and included those associated with travel, other commitments and a general lack of interest in the activity. One teacher provided some insight into the low rates of transition into the community programs suggesting, *“It’s probably because nothing has ever really been offered here at this school before…. It’s a whole new concept….”* (PE Teacher, Intervention School H).

### Implementation: extent and consistency of program delivery and associated implementation issues

#### The school component

Teachers provided feedback (n = 79 classes; 60.8% response rate) on the extent to which the lesson plans contained within the teaching manual were implemented. Teachers reported that the lesson was implemented in line with the lesson plan 'almost all’ (n = 176 lessons; 54.3%) or 'most’ (n = 79; 24.4%) of the time. However, focus group discussions with teachers and coaches revealed that the understanding of, and commitment to, the intention of a Game Sense approach varied and subsequently influenced the consistency of program implementation. Firstly, there were varying degrees to which teachers and coaches subscribed to the intentions of a Game Sense approach [42]. Specifically, performance discourses [43] tended to be emphasised by teachers and coaches, whereby skill, technique and competition were privileged. For instance, some teachers and coaches commented that they incorporated skills and drills into lessons as they felt students’ fundamental motor skills were low. As a consequence, lessons were at times re-structured to include a warm up, followed by skill/drill and concluded with a game, which is aligned with traditional pedagogies based upon performance discourses that reinforce the acquisition of skills through demonstration, explanation and practice teaching approaches [44]. The following passages highlight the perception among some teachers that fundamental motor skills were a prerequisite to game play:

So okay, it was all on this game-sense stuff but if you couldn’t kick accurately to this person or it’s gone out of bounds, oh well come back and we’ll start again … by year nine if you’ve never really played [tennis], you are a long way behind those that do play so lots of the rally type things, hitting into this box or some of the kids had no hope of even getting it in that direction, if at all over the net… (PE Teacher, Intervention School G)

In addition, some teachers had little or no experience with Game Sense; they discussed the approach as being quite new, reflecting that they were unsure on their execution of the approach (e.g. *“I try to do it, I don’t know how well I do it, but I try”* PE Teacher, Intervention School E). Another explained that they were from the *“older methods”* of teaching and despite some professional development on Game Sense, found it difficult to *“take the next step to actually put it into your life, I think you need to revisit it again and again and again before you actually get that change happening….”* (PE Teacher, Intervention School D). Similarly, some coaches were more experienced than others with Game Sense pedagogy, as some commonly applied it to their coaching practices, whilst others had not. Experienced coaches commented that Game Sense was ideal for girls who had limited fundamental motor skills, enjoyed the social nature of participation and who did not want to focus on competition or technical aspects of the sport. These comments from teachers and coaches highlight the need for ongoing dialogue with the research team during implementation to help overcome the challenges associated with implementing alternative teaching pedagogies.

There were also some communication breakdowns between the schools, coaches and instructors, which resulted in a small number of school sessions being rescheduled or cancelled. For example, one tennis coach commented that one school’s *“human resource and organisation was non-existent and their [the school’s] compliance to the details and structures that were set out were non-existent”* (Tennis Coach). It was felt that the school’s lack of commitment hindered the coach’s ability to deliver the program successfully. Another coach reported that classes were often late to program sessions. In addition, the length of PE classes ranged from 60 to 100 minutes across the seven schools, and as such coaches commented that 60 minutes was often not enough time to deliver the content and 100 minutes was too long to keep students engaged. However, leisure centre instructors felt that even 60 minutes was too long, particularly since their community-based programs such as group fitness classes are structured around 45-minute sessions.

#### Facilitating outside-of school participation

The intervention incorporated several elements to promote PA outside of school including the self-management journal activities to encourage girls to be independently active and also formally linked PE classes with existing community sport and recreation providers. In terms of the extent that the self-management journal activities were implemented, teachers reported, that the majority were not implemented at all (n = 203; 60.2%), although a third of teachers set the journal activities as homework (n = 108; 32.0%). The main reason that journal activities were not implemented was that teachers prioritised the PA component of the program over the cognitive component during class time. As a result, very few students reported that self-management journal activities were completed; indeed the majority were unaware of these activities. There were, however two cases in which groups of students discussed the journal activities that their teacher had set and they had commented that they *“jumped over pillows”* when they designed a fitness circuit at home or *“started walking the dog”* to fit physical activity into their lives outside of school (Intervention School E, Year 7).

PE teachers, coaches and instructors attempted to encourage students to attend outside-of-school programs by using strategies such as flyers, notices in school newsletters and 'spruiking’ during PE classes. Students who had completed the baseline surveys also received SMS messages generated from the research team. There were a few instances, however, where community programs were not discussed appropriately or in a timely manner with students by the coaches or instructors. In these instances, students were provided only with vague details about the programs, due to a lack of communication between the coach/instructor within the school and the local club program facilitator. For example, a teacher reflected:

With the football coach there was no talk of come down, … it was driven by me and I thought here is your opportunity mate to get kids to be excited and come down to your club and build your numbers and that didn’t come through at all. (PE Teacher, Intervention School D)

Another factor impacting upon the uptake of the community programs was that while it was envisaged that students would be taken to visit the community clubs and leisure centre facility at least once during each unit, not all participating PE classes did so. In one case the facility was fully booked for public programmed classes and in another three cases there was poor communication between the coach and the community club. As a consequence, some students were not exposed to the clubs/facilities as intended, which is likely to have impacted on the likelihood of attending the subsequent community program.

### Satisfaction with the program

#### Student engagement and learning

Student response to the lessons was reported by teachers as positive or very positive (n = 278; 80.3%), although Year 9 students were reported to respond less positively for both football and tennis lessons. Teachers also reported that students responded positively or very positively to the coaches’ methods/approach/style (n = 178; 86.0%).

The qualitative focus groups further explored participants’ satisfaction with the program and the associated factors that influenced program satisfaction. Primarily, teachers were satisfied with the program because they perceived that PA behaviour among the girls improved where *“even the most inactive girl was out there having a go”* in the PE classes (PE Teacher, Intervention School F). Teachers noted that participation improved since the emphasis of the program was on games rather than skills and drills, included recreational activities via the leisure centre program, and had opportunities for single-sex classes. Teacher commented they enjoyed it because:

They weren’t drills. Kids hate drills. Absolutely hate them. My kids are like, do we have to do drills but with the game-sense model I found it worked way better” (PE Teacher, Intervention School C).

“They get lost in the moment of doing a body attack, doing a pump class and they could just go for it” (PE Teacher, Intervention School D).

Some teachers also reported that students’ confidence in activities increased and were surprised at the level of skill developed during the school component, although some teachers also commented that the level of skill development was probably not enough for students to confidently transfer their participation to organised sport outside of school. Teachers attributed the increase in confidence and skill development to the inclusion of activities that focused on making small skill progressions and more specifically, enhancing cognitive understanding of the tactics and strategies that underpin game participation, which was a fundamental principle of the Game Sense approach.

Students also reported enjoying the activities and teaching approach adopted in the design of the program. They commented that they learnt new sports skills, such as learning how to serve and rally in tennis, and dribble and score goals in football. In particular, students who perceived themselves as “not sporty” reported feelings of success in performing sports activities and as such reported the development of new skills. For example students commented:

[I learnt] a lot seeing as how I don’t really play any sports so it was quite nice to actually be able to play a sport and not completely suck at it (Intervention School D, Year 7).

Yeah, like it [the teaching/coaching] shows you how to do it. You’re not just playing and going, what do I do….and you don’t just kick the ball…..we learned skills and then he [the coach] said that we have to do that sometime in the game, and you get a point that you do that. So it showed us how to do it [the skill in a game-based situation]. (Intervention School C, Year 9)

Students also reported learning about the availability of different types of sports and physical activities in the community. In particular they commented that they enjoyed the recreational activities organised by the leisure centres, which were commented on positively for their ability to offer something 'different’ from the usual PE lesson that in our participants’ experience tended to privilege team sports and games. For instance, a student reflected on her experience with some of the leisure centre activities which included Zumba and fitness circuits:

I liked the change in what we were doing [with leisure centre activities] because when you do sports at school, it’s always just these games you have to learn how to play when you didn’t want to, but there you learned something different than usual; [it] was actually good to learn and could always help you later in life. (Intervention School D, Year 8).

The inclusion of single-sex classes was a topic hotly debated by girls. A number of girls who perceived themselves to be either 'sporty’ or 'non-sporty’ and either active or non-active reported that they enjoyed single-sex classes because they had *“a more even chance”* of playing and being active in the PE class; especially because boys were not dominating the activity, teasing, or being too competitive. One student reflected that the girls-only classes were important *“…because most of the girls that do PE with boys, they just don’t do it. They don’t bring their uniforms and then they don’t do it.”* (Intervention School H, Year 7). This comment was reiterated by a student at another school who commented *“…and girls weren’t embarrassed at all. Like the girls who sit out they were happy to join in and they had a lot of fun”* (Intervention School G, Year 10).

On the other hand, co-educational classes were preferred by some girls who had experienced negative peer interactions with other girls – *“Most of the girls were harder to make friends with, and like be social with, like they all have their own groups of friends; whereas the guys just blend in and then you can blend in too. Girls just seem to stick to their own little groups.”*(Intervention School D, Year 9). Co-educational PE classes were also preferred by some sporty or active girls, as boys provided the opportunity to have *“someone to compete against”* (Intervention School D, Year 8), whereas less sporty or active girls were more likely to minimise their participation.

There was also a lack of agreement among teachers, coaches and instructors about the perceived benefits of single-sex classes. Some teachers reported that their students enjoyed the opportunity for single-sex classes; however others reported that single-sex classes did not work and suggested that cohesion and inclusiveness of the students within the class was more important than the gender mix. Tennis and football coaches, however, consistently reported that single-sex PE classes worked better than co-educational classes. They commented *“boys just upset the class totally....when the girls were by themselves, absolutely fantastic.”* (Tennis coach). It seems that coaches found the delivery of single-sex classes easier to implement as they did not need to spend as much time managing students in single-sex classes.

#### Capacity building strategies

Professional development opportunities and resources were provided to teachers, coaches and instructors to support program implementation. A total of 45 teachers from the seven schools participated in professional development workshops conducted at each school by research staff. The aim of the workshop was to 1) present to teachers a range of activities that model a Game Sense approach to teaching tennis and football; 2) to present fitness activity ideas and discuss these activities in terms of technique, safety, progressions and other relevant considerations; and 3) build partnerships between teachers the community sport and recreation providers. Workshop feedback was received from 41 teachers (91.1%) and was generally very positive. Teachers mostly agreed or strongly agreed that the content was interesting and relevant (n = 38; 92.7%), that the time and duration of the workshop was appropriate (n = 39; 95.1%), and that participants had an opportunity to contribute to discussion (n = 40; 97.6%), and to ask questions or clarify issues (n = 40; 97.6%). Participants also agreed or strongly agreed that as a result of participating in the workshop they felt more aware of the key issues for girls regarding participation in PE, community-based sport and PA (n = 35; 86.4%).They also reported that the workshop confirmed some of their own perceptions about the issues girls face in terms of PA participation (n = 37; 92.5%). As part of the workshop, activities were conducted with teachers to identify how each school could create more meaningful and relevant learning experiences for girls in PE and to plan how the program could be implemented within the existing school structure. Teachers agreed or strongly agreed that both of these activities were useful (n = 38; 92.7% and n = 36; 87.8% respectively). Overall teachers rated the workshop as good (n = 12; 29.3%) or very good (n = 26; 63.4%).

The focus group discussions further supported the notion that teachers were satisfied with many of the resources provided; particularly the lesson plans which they reported challenged students to participate in activities because it focused on games rather than drills and higher order thinking through questioning. It also provided teachers with flexibility to respond to the needs of the students. For example a teacher commented:

Loved the questioning, loved the variety of activities and I suppose the flexibility of it…. If it wasn’t working, then you use your own knowledge, your own assessment of the kids to modify from there and if one activity didn’t work you could hopefully go on to a different one….You did have some substance in activity and questioning and resources behind it and I think that was valuable. (PE Teacher, Intervention School D)

A number of teachers, however, indicated that there were school timetable constraints on implementing the program to its full potential, and felt that PE was often marginalised within the broader school curriculum. Teachers commented that it was difficult to provide positive experiences in PE for students when PE was marginalised in the school curriculum. For example, a group of teachers commented:

Unfortunately I don’t think our school or our administration values the PE or the sport or health very highly…..but then they’re telling [the sports coordinator] that the culture needs to change in sport …[or]…they won’t be spending any more money…[but]…they don’t listen – that to change [the sport] culture you need to change it in PE first. You’ve got to change it there for kids who want to go further and do more stuff… At the moment we don’t even have two basketball rings working properly. One’s broken and they’ve just tied it up so now we can’t play basketball in the gym. (PE Teacher, Intervention School C)

Coaches and instructors also reported being mostly satisfied with the resources such as the lesson plans provided to implement the program. However there was a lack of capacity among some providers to implement activities in PE classes; particularly among leisure centre providers. First, there was a lack of experience or capacity among some leisure centre instructors to implement recreational/leisure activities for secondary school students whose motivation, interest, and fitness levels varied. For example, some leisure centre instructors were reported by teachers to be unable to cater to the students’ fitness levels and create a supportive class room environment on which to build student learning and interest in recreational activities. One teacher described an instructor as very militant, using a command style and explained *“I was there for one class and these kids were working hard, really hard, she was like 'you’re not working hard enough! Come on kids!’ I was like 'ease up!’”* (PE Teacher, Intervention School B). Second, some leisure centre facilities did not have the staffing capacity to implement a wide range of recreational activities; therefore students only received a somewhat limited scope of activities in their leisure centre-based lessons, which tended to be focused on fitness circuits rather than incorporating other activities that leisure centres have available. Third, the group fitness room at one leisure centre facility had clear windows and doors and therefore participants were visible to the public, which made students self-conscious about people watching and resulted in students disengaging from the activity.

## Discussion

The implementation of the program aimed to make positive changes to rural and regional-living adolescent girls’ health-related quality of life (HRQoL), PA levels and mediators of PA. The program was implemented in a real world setting, with PE teachers engaging in professional development opportunities that sought to introduce them to the principles underpinning the design of lesson plans and facilitate links to community-based sport and recreation providers. There were a number of significant positive results in terms of program effectiveness [10], which were influenced by implementation factors related to the capacity building framework adopted in this study including satisfaction with professional development opportunities, including the training, resource manual and opportunities to work with coaches and instructors during PE classes. Furthermore, a number of positive perceptions from students and teachers were reported in relation to the program design which supported student learning and engagement. In particular, students reported increased awareness of PA opportunities outside of school, the development of new sport specific and PA skills and enjoyment of the program. Students also talked about having an understanding of when certain strategies would be used in game situations demonstrating the development of higher order thinking skills.

However, notwithstanding some positive outcomes, the degree of success of the intervention was limited by a range of factors. This study provides critical discussion around the barriers to implementation of a school-community linked program to inform practitioners and future intervention-based research in schools.

The socio-ecological model was a useful framework to consider and address the wide range of factors that influence PA behaviour and as such the program was underpinned by several elements reported to be most effective in promoting PA among girls [3, 6]. These elements included: a multicomponent intervention with school and community opportunities for PA, addressing multiple levels of influence on behaviour, and applying student-centred pedagogical approaches and self-management strategies to engage students in a traditional team sport (football), a lifestyle sport (tennis) and a range of lifestyle physical activities (leisure centre activities) both in PE and in the community. The intervention, however, did not include a focus on family support and instead focused on inclusive peer strategies. Whilst family support has been reported as ineffective by some [3], others have reported a positive impact of family support on sports club membership of adolescent girls [45]. In particular, family support may be important in a regional context, especially where public transport is limited, to ensure young people can access PA opportunities outside of school [45]. This study found that students experienced barriers related to travel including getting to and from PA opportunities. Strategies to support young people and their families may be required, particularly in low socioeconomic communities where access to familial resources such as a parent’s ability to make themselves available to transport children to/from opportunities to engage in PA are hampered [24].

The likelihood of such a program achieving the intended health outcomes, such as changes in HRQoL, PA or mediators of PA is highly dependent on its implementation. Schools are undoubtedly a complex and challenging setting in which to implement change and are often constrained by barriers such as organizational factors, policy constraints, lack of resources and a crowded curriculum [12]. More specifically, difficulties associated with implementing interventions in schools have included the: lack of teacher adoption, lack of program readiness, absence of program champions (advocates), inadequate or unsustainable funding, inadequate infrastructure, poor integration of the program within existing structures and programs, limited teacher training, insufficient program support materials and staff turnover [31]. In this study, implementation was sometimes negatively influenced by a lack of individual and organisational readiness to adopt program principles. This was despite the apparent positive response to the individual-focused strategies implemented under the capacity building framework.

Individual and organisational readiness to implement the program varied. For instance, self-management strategies were not well implemented by teachers because they tended to implement cognitive components of the intervention in a selective manner. Furthermore, there were deeply embedded ideologies based upon performance discourses that reinforce the acquisition of skills through demonstration, explanation and practice teaching approaches [44], which was in contrast to the principle of Game Sense, which seeks to refocus attention from skill performance to the tactical and strategic (cognitive) dimensions of a game. The Game Sense model was purposefully adopted for the sports of football and tennis to reduce the focus on skill performance in PE settings as raised by adolescent girls in the ethnographic fieldwork phase of the study [22]. It was also selected because Game Sense aligned with recent shifts in both sports coaching and the teaching of games [46].

Teaching pedagogies such as Game Sense often incite resistance from practitioners because they challenge the traditional and enduring belief that fundamental movement skills are prerequisite to game play [44]. The implementation of Game Sense is often fraught with challenges including the change in decision making from the teacher to the student, variations in the interpretation of Games Sense [47], lack of exposure to effective game-based professional development opportunities, and performance discourses embedded within PE and youth sport programs [48]. Game Sense approaches have reported opportunities for student decision making, social interaction and cognitive understanding and/or high order thinking [49]. Further, higher levels of enjoyment, particularly among girls has been reported in approaches that draw on Game Sense principles [50]. Whilst capacity building strategies to empower teachers and coaches to implement a Game Sense approach, as well as other elements of the intervention like self-management strategies, were provided further work is required. It appeared that teachers and coaches would select aspects of the lessons that resonated with their own theories of practice. This may have occurred as their professional development (in relation to the program) was not necessarily ongoing or sustained, rather it was limited to a one day professional development session supplemented with collaborative learning opportunities with the introduction of coaches and instructors into the PE setting. It has been suggested that professional development for PE teachers has greater impact when it is ongoing and teachers place high value on learning collaboratively with and from each other in professional learning communities or networks [51]. Therefore, ongoing sustained opportunities to engage in critical reflection throughout implementation of the intervention and/or provision of digital resources that support implementation (e.g. case studies and video clips that describe some of the challenges of using a Game Sense approach) is important to further build individual capacities in future school-focused and pedagogical-based PA interventions. Particularly, to realise the potential of Game Sense and broader game-based approaches to enhance girls participation in PA [42].

Fundamentally, despite the use of a range of capacity building strategies, the implementation of the program required organizational changes in order to maximise individual changes at the student level. Weiner [52] conceptually theorized organizational readiness as “a shared psychological state in which organizational members feel committed to implementing an organizational change and confident in their collective abilities to do so” (p.1). Further Weiner [52] states,

Organizational readiness for change varies as a function of how much organizational members value the change…When organizational readiness for change is high, organizational members are more likely to initiate change, exert greater effort, exhibit greater persistence, and display more cooperative behavior. The result is more effective implementation.

Organizational readiness for change has been identified as an under-recognised area in health promotion practice [53] and there is a need for greater recognition of the time it takes to facilitate organizational change, particularly within schools, to support the implementation of health promoting programs [54]. However, organizational readiness and capacity for change has not been subjected to extensive theoretical development and empirical study [52]. Considering the varying understandings of, and commitment to, the intention of a Game Sense approach and the cognitive component within this study; along with a perception that PE was marginalized within the wider school curriculum, understanding individual and organizational readiness may be especially important to determine the shared commitment and ability of program deliverers to implement the program. As such, future interventions and research studies should seek to assess and develop individual and organizational readiness within the study design to better engage schools and sport and recreation providers in the provision of school and community linked PA opportunities for adolescent girls.

Single-sex classes were a contentious issue in this study. Since the mid 1980’s there has been a shift in Australia to the implementation of co-educational classes for PE; although there is continuing controversy on co-educational and single-sex PE [55, 56]. As reported in previous research, girls often need to feel emotionally safe to fully participate as boys often dominate play in game-situations [57] and trivialise girls’ interests, concerns and physical behaviours [58]. Students construct meaning from their experiences in PE lessons by drawing upon their thinking about many factors including their bodies, gender and societal norms. It has long been argued that the dominant masculine practice of traditional team sports in the PE curriculum and has tended to privilege boys and marginalised girls [59]. Further, the role of the teacher in the construction and reproduction of, and resistance to dominant gender power relations has been of interest to educational researchers for some time [59, 60]. Teachers expect girls to be tentative and reluctant to reaffirm their femininity; whereas, boys are assumed to be more compliant, because for a boy to display resistance in PE would question his masculinity [59]. Various strategies were suggested within the lesson plans to emphasise inclusive interactions among students regardless of how the PE classes were organised (single sex or co-educational). In this study, schools tended to implement single sex classes to deliver the program, which was received positively for a number of students. However, it appeared that efficient skills in managing group dynamics were important. The impact teachers, coaches and instructors can make on students’ social interactions within the PE, sport and recreation setting remains an area warranting further study.

Some limitations to this study need to be acknowledged. Direct observation of program implementation may have strengthened the assessment of process evaluation; although this too is not without measurement issues. Direct observation was not practical for this large study due to the high cost associated per observation [61]. In addition, the presence of observers may affect the implementation behaviour of program deliverers and/or student behaviour [61]. Instead, data triangulation was implemented which involved teachers completing a short feedback form after each class to capture immediate thoughts and reflections and later teachers participated in a focus group to provide an overall reflection on the program. The response rate for the feedback forms was moderate (60%) and attendance by teachers at focus groups was high (>85%), which was useful to generate meaningful discussion about their experiences. Upon reflection of the evaluation plan, data triangulation was a useful method as the feedback forms indicated a high level of program implementation, although focus groups revealed that the understanding of, and commitment to, the intention of a Game Sense approach varied and subsequently influenced the consistency of program implementation. In future evaluation plans, an online reporting system, with all registered program deliverers sent email reminders may improve recording of immediate feedback.

## Conclusion

Reach, adoption and implementation were high due to the provision of professional development opportunities and resources to deliver the program and satisfaction with the design of the program by teachers, coaches/instructors and students. It was evident, however, that some aspects of the program were not fully implemented as intended, which affected the likelihood of the program “quantitatively” achieving other intended public health outcomes, such as improvements in PA levels. This study provides useful information for future school-community linked interventions to address barriers to implementation; in particular the need to assess and control for organizational readiness within study designs.
